# Epigenetic silencing of ZIC4 contributes to cancer progression in hepatocellular carcinoma

**DOI:** 10.1038/s41419-020-03109-1

**Published:** 2020-10-23

**Authors:** Wenbiao Chen, Donge Tang, Dongxin Tang, Yong Dai

**Affiliations:** 1 Department of Clinical Medical Research Center, The Second Clinical Medical College of Jinan University, The First Affiliated Hospital Southern University of Science and Technology, Shenzhen People’s Hospital, Shenzhen, 518020 China; 2 Department of Science and Education, The First Affiliated Hospital of Guiyang University of Chinese Medicine, Guiyang, 550001 Guizhou China

**Keywords:** Cancer therapy, Tumour angiogenesis, Cell migration

## Abstract

Inactivation of tumor suppressor gene played critical roles in the development and progression of human hepatocellular carcinoma (HCC). Zic family member 4 (ZIC4) is transcription factor and plays an important role in the developmental process. However, the expression and biological role of ZIC4 in HCC is poorly understood. Here, bioinformatics analysis based on The Cancer Genome Atlas (TCGA) database revealed an aberrant hypermethylation of ZIC4 in HCC. ZIC4 is frequently hypermethylated in promoter region and down expressed in HCC cells and tissues. Functionally, ZIC4 inhibition facilitated the proliferation, migration, invasion, and epithelial-mesenchymal transition (EMT) in vitro and in vivo. Conversely, ZIC4 overexpression reduced proliferation and invasiveness of HCC cells. In addition, ZIC4 inhibition rescued the antitumor effect induced by enhancer of zeste homolog 2 (EZH2) knockdown or EZH2 inhibitor. Mechanistically, EZH2 knockdown or EZH2 inhibitor reduced the enrichment of EZH2 and H3K27me3 in ZIC4 promoter region and leading to the upregulation of ZIC4. Altogether, these data indicate that epigenetic silencing of ZIC4 by EZH2 mediated H3K27me3 is an important mechanism in HCC and provide a new therapeutic target for the treatment of hepatocellular carcinoma disease.

## Introduction

Liver cancer is the fifth most critical malignant tumor and the second common leading cause of cancer-related death in males, according to statistics, there are about 782,500 people were diagnosed with liver cancer and 745,500 deaths in 2012 all over the world^1^. Hepatocellular carcinoma (HCC) has poor prognosis and is one of the most common type of liver cancer^2^. Many studies have reported that the etiological causes of HCC vary from birth and area, such as intake of alcohol in western countries, hepatitis B virus infection in Africa and East Asia, the infection of hepatitis C virus in Japan and the exposure of aflatoxin B1 in Africa and China^3^. However, the decided molecular biological mechanisms that cause HCC development are still unclear, recent data suggest that the incidence of HCC is a complex process, including the activation of proto oncogenes and the inactivation of tumor inhibitor genes caused by abnormal epigenetic and genetic alterations^4^. Nevertheless, none of this information has been applied to clinical. Consequently, a deeply understanding of HCC oncogenesis and development is important to recognize corresponding molecular therapeutic mechanisms.

Epigenetic mechanisms such as histone modifications, DNA methylation, non-coding RNAs and chromatin remodeling were proved to regulate gene expression by many cross talk ways^5^. DNA methylation is one of the most widely studied epigenetic mechanisms, which is a covalent modification of DNA, plays an important role in the regulation of genome function, often occurs in hepatocellular carcinoma, bladder cancer, prostate cancer and many other common cancers^6^. It was confirmed that aberrant DNA methylation of promoter CpG islands was involved with the tumor inhibitor genes silenced in many types of human cancer-related diseases^7^. For example, Sun et al. illustrated that tissue suppressor of metalloproteinases3 (*TIMP-3*), cyclooxygenase2 (*COX2*), ras association domain family 1 isoform A (*RASSF1A*) and *p16* genes are usually hypermethylated in HCC, but the methylation not occurred in non-tumor liver tissues^8^. Therefore, we proposed that DNA methylation might involve with HCC progression.

As we all known that DNA methylation is suppressed by DNA methyltransferases inhibitor such as 5-Aza-2′-deoxycytidine (5-aza-dC)^9^. Nowadays, the studies on the anti-tumor molecular mechanisms ground on epigenetic drugs are developing, 5-aza-dC has been allowed in the clinical treatment of haematological malignancies and its influence on solid tumors were worth investigation^10^. Anwar et al. found that 5-aza-dC treatment could decrease the methylation degree and restored the expression of miR-183^11^. EZH2, which is a histone methyltransferase, also plays a crucial role in many different kinds of cancers via epigenetic silencing of tumor inhibitor genes^12^. Previous studies showed that 3-deanzaneplanocin A (DZNep), which is an inhibitor of S-adenosylhomocysteine hydrolase, could deplete PRC2 components cellular level, such as SUZ12, EED, and EZH2, and further suppress the H3K27 trimethylation^13^. Therefore, we hypothesized EZH2 might involve HCC progression via H3K27me3 pathway.

It is well known that *ZIC* gene family function as a vital role in the development of neural crest and subsequent cerebellar^14^. Previous studies showed that *ZIC* gene family involved with cell proliferation regulation via suppressing neural differentiation in the dorsal neural tube, however the exact biochemical and cellular mechanism are still unknown^15^. Kandimalla et al. reported that the methylation of GATA binding protein 2 (*GATA2*), T-box 2 (*TBX2*), T-box 3 (*TBX3*), and Zic family member 4 (*ZIC4*) genes were related to the development of muscle invasive disease of papillary non-invasive bladder tumors^16^. Our study aimed to investigate the effect of *ZIC4* hypermethylation on HCC progression both in vitro and in vivo.

In the present study, we investigated DNA methylation expression profiles of HCC tumor and adjacent tissues. The interactions among EZH2, H3K27me3, and *ZIC4* promoter was studied, and the biological function of EZH2 and ZIC4 on HCC progression also has been explored both in vitro and in vivo. The results also pointed that epigenetic silencing of *ZIC4* by EZH2 mediated H3K27me3 is an important mechanism in human liver cancer, which are very important for subsequent clinical research.

## Materials and methods

### Clinical samples

This study was approved by the institutional review board of Shenzhen People’s Hospital ethics committee and informed consent was obtained from all patients included in this study. A total of 20 hepatocellular carcinoma patients with pairs of the tumor and the adjacent normal tissues were recruited from Shenzhen People’s Hospital, which were histologically or cytologically confirmed by at least two local pathologists. Tissues were frozen in liquid nitrogen after the surgery and stored at −80 °C.

### Bioinformatic analysis

The genome-wide methylation data of an independent dataset (The Cancer Genome Atlas) consisting of 50 HCC samples and their matched surrounding tissues were employed. The Chip Analysis Methylation Pipeline (ChAMP) package is a pipeline which not only integrates currently available 450k analysis methods but also offers its own novel functionality. Statistical analyze of DNA genome methylation profile was performed on Illumina BeadStudio software (Genetech Biotech, Taipei, Taiwan). Β values were calculated during this procedure, obtained results were selected from 0 to 0.1 to represent CpG loci, and 0 to 100% on behalf of the percentage of methylation, respectively.

### Cell culture and drug treatment

The human HCC-derived Hep3B, Huh-7 and HepG2 were obtained from the BeNa Culture Collection (Shanghai, China). LO2 cell line was acquired from Procell Life Technology (Wuhuan, China). All cell lines were authenticated using short tandem repeat (STR) profiling at the time of purchase. Low-passage cells were used for experiments within a period of 6 months after resuscitation. All cells were negative for mycoplasma contamination. All cell lines were cultivated cultured routinely in Dulbecco’s modified Eagle’s medium (DMEM) supplemented with 10% heat-inactivated fetal bovine serum at 37 °C in a humidified 5% CO_2_ incubator. DZNep was purchased from MedChemExpress (MCE; Monmouth Junction, NJ, USA) and dissolved in ddH_2_O. 5-aza-2′-deoxycytidine was purchased from Sigma-Aldrich (St. Louis, MO, USA) and dissolved in DMSO. For the 5-Aza-dC treatment, 5-Aza-dC (5 μM) was replenished daily for 72 h. For the DZNep treatment, DZNep (10 μM) was added to the culture medium for 72 h.

### Transfection of shRNA

The shRNA against EZH2 and ZIC4 were synthesized by GenePharma (Shanghai, China). Transient transfections were performed by Lipofectamine 3000 cell transfection agents (Invitrogen, Carlsbad, CA, USA) following the manufacturer’s protocol. The sh-RNA sequences are listed in Supplementary Table 1.

### DNA methylation analysis

DNA was isolated using the proteinase K/phenol extraction method. Bisulfite conversion was carried out using 1 μg of DNA using an Epitect Bisulfite Kit (Qiagen). Bisulfite-treated DNA was amplified with BSP primers located in the *ZIC4* promoter and PCR products were cloned using the pGEM-T Easy Vector system (Promega, Madison, WI). Three individual clones were sequenced. The region assessed by BSP included 8 CpG sites from the *ZIC4* promoter and average methylation from individual clones was calculated as a percentage of the number of methylated CpG sites over the number of total CpG sites sequenced.

### RNA extraction and quantitative reverse transcription-polymerase chain reaction (qRT-PCR)

Total RNA from frozen tissue specimens and cultured cells was extracted using TRIzol reagent (Invitrogen, Carlsbad, CA) according to the manufacturer’s instructions. RNA quantity and quality were determined by a NanoDrop ND-1000 Spectrophotometer (NanoDrop Technologies, Wilmington DE). The cDNA was synthesized from total RNA using PrimeScript™ RT Reagent Kit with miRNAs specific RT primers (Takara, Dalian, China). Real-time PCR was performed on the Applied Biosystems 7300 Real-Time PCR system using SYBR Green dye (Applied Biosystems, Foster City, CA) as described by the manufacture. The relative expression levels were evaluated by using the by $2^{-\Delta\Delta C_t}$ method. Primers for each gene were listed in Supplementary Table 1. All results were expressed as the mean ± standard deviation (SD) of three independent experiments.

### Western blotting

Western blotting was performed in cultured cells as indicated. After the cells were lysed in buffer containing 1% NP40, 50 mM Tris, 5 mM EDTA, 1% sodium deoxycholate, 1% SDS, 1% Triton X-100, 10 mg/ml aprotinin, 1 mM PMSF, 1 mg/ml leupeptin, and pH = 7.5, supernatants were collected after spin and protein was measured by Bradford assay (Thermo, Waltham, MA, USA). Lysis were resolved by SDS–PAGE and transferred to a PVDF membrane (Millipore, Billerica, MA). The blots with proteins were then blocked by 5% non-fat dry milk and incubated with appropriate primary antibodies at 4 °C overnight. The membranes were then incubated by HRP conjugated secondary antibody, and signals were visualized by an enhanced ECL-based imaging system. Antibodies used in the study include anti-ZIC4 (ab178512), anti-β-actin (ab179467), anti-EZH2 (ab191250), anti- H3K27me3 (ab192985), E-cadherin (ab1416), N-cadherin (ab76057), Vimentin (ab92547), goat anti-rabbit IgG H&L (HRP) (ab6721) and rabbit anti-mouse IgG H&L (HRP) (ab6728).

### Chromatin immunoprecipitation assay

ChIP assay was performed using the EZ-CHIP chromatin immunoprecipitation kit (Millipore, Billerica, MA) following the manufacturer’s protocol. Immunoprecipitate (IP) complexes were immunoprecipitated with an anti-EZH2 (ab191250), anti-H3K27me3 (ab192985) antibodies or rabbit IgG antibody overnight at 4 °C. The captured genomic DNA was obtained and used for quantitative PCR analysis. Ten per cent of total genomic DNA from the nuclear extract was used as input. Amplification efficiency was calculated, and the data were expressed as enrichment related to input.

### Wound healing and transwell assays

For wound healing analysis, 24 h after the transfection, the cells were plated in 6-well plates. 24 h later, the adherent cells were wounded by a 10 μl plastic pipette tip. Then rinsing the scathing cells with PBS and culturing with serum-free DMEM for 24 h or 48 h. The wound closure in different groups was photographed and evaluated with the microscope. For the transwell assays, 1 × 10^5^ cells in medium containing 0.1% FBS were seeded into the upper chamber with or without a matrigel (1:30 dilution) coated membrane (BD Biosciences, Franklin Lakes, NJ), while medium containing 10% FBS was in the under chamber. After incubation at 37 °C, gel and cells in the upper chamber were removed carefully and cells adhering to the underside of the membrane were stained with 0.1% crystal violet (Beyotime Institute of Biotechnology) and 20% methanol. The numbers of cells were counted under an inverted microscope (Nikon). All results were expressed as the mean ± SD of three independent experiments.

### Colony formation assay

24 h after the transfection, HepG2 cells were resuspended and seeded onto 12-well plates at a density of 2000 cells/well, incubated for two weeks, and then stained with 0.5% crystal violet for 30 min. Excess dye was rinsed off twice with phosphate-buffered saline (PBS). Images were obtained using the computer software Quantity One® from Bio-Rad Laboratories, Inc. Colonies containing more than 50 cells were counted. All results were expressed as the mean ± SD of three independent experiments.

### Flow cytometric assays for apoptosis

After digested and washed twice with ice-cold PBS, cells were collected and resuspended with binding buffer. Cell staining was performed using AnncxinV-FITC Apoptosis Kit (Beyotime, Shanghai, China). Finally, the FACSCalibur flow analyzer (BD, CA, USA) was used to complete the detection within 30 min. All results were expressed as the mean ± SD of three independent experiments.

### HCC mouse model

Four- to five-week-old female BALB/c nude mice were obtained from the Animal Center of the Chinese Academy of Medical Sciences (Beijing, China). Mice age 5–6 weeks were injected subcutaneously in the flank with 1 × 10^7^ HepG2 cells with or without ZIC4 stably knockdown and were randomly divided into four groups (5 mice/group). 1 week after bearing, DZNep (1 mg/Kg) was administered intraperitoneally twice per week and every 2 weeks. Tumor size was monitored by digital caliper. Tumor volume = (L × W^2^)/2, where L is length at the widest point of the tumor and W is the maximum width perpendicular to L. Solid tumors were harvested, fixed with phosphate-buffered neutral formalin, sectioned serially and stained with hematoxylin and eosin (H&E) and EZH2, ZIC4, Ki67, E-cadherin and N-cadherin IHC staining for standard histological examination. The subcutaneous tumor tissues were removed and implanted into the liver of nude mouse to conduct the orthotopic implantation (5 mice/group). DZNep (1 mg/Kg) was also administered intraperitoneally twice per week and every 2 weeks. After 6 weeks, the metastases were visualized using the IVIS@ Lumina II system (Caliper Life Sciences, Hopkinton, MA) 15 min after intraperitoneal injection of 3.0 mg of D-Luciferin potassium salt (Sigma-Aldrich, St. Louis, MO, USA) in 200 µl of sterile PBS without magnesium or calcium. Lungs were harvested and H&E staining was performed. The metastatic nodules in each lung were calculated. All animal procedures were approved and performed in accordance with the guidelines of Shenzhen People’s Hospital ethics committee. Standard of blinding and randomization was complied with in this study.

### Statistical analysis

SPSS 19.0 statistical software (Chicago, IL, USA) was used for statistical analysis. Quantitative data were expressed as mean ± SD. A comparison of groups was analyzed by single factor ANOVA and qualitative data were expressed as the number of cases or percentage (%). A comparison of the groups was made using the χ2 test. Statistical significance was set at *P* < 0.05.

## Results

### *ZIC4* was hypermethylated in HCC and correlated with survival

The genome-wide methylation data of an independent dataset (The Cancer Genome Atlas) consisting of 50 HCC samples and their matched surrounding tissues were employed and found that *ZIC4* was hypermethylated in tumor tissues compared to normal tissues in the top 30 candidate genes (Fig. S1A). Higher *ZIC4* methylation in tumor tissues compared to paired normal tissues was displayed (Fig. S1B). Kaplan–Meier analysis showed higher methylation of *ZIC4* group involved with shorter survival rate (Fig. S1C). Altogether, these results suggested that *ZIC4* was hypermethylated in HCC tissues and involved with poor survival rate.

### Loss of *ZIC4* occurs in HCC with epigenetic abnormalities

**Fig. 1:**
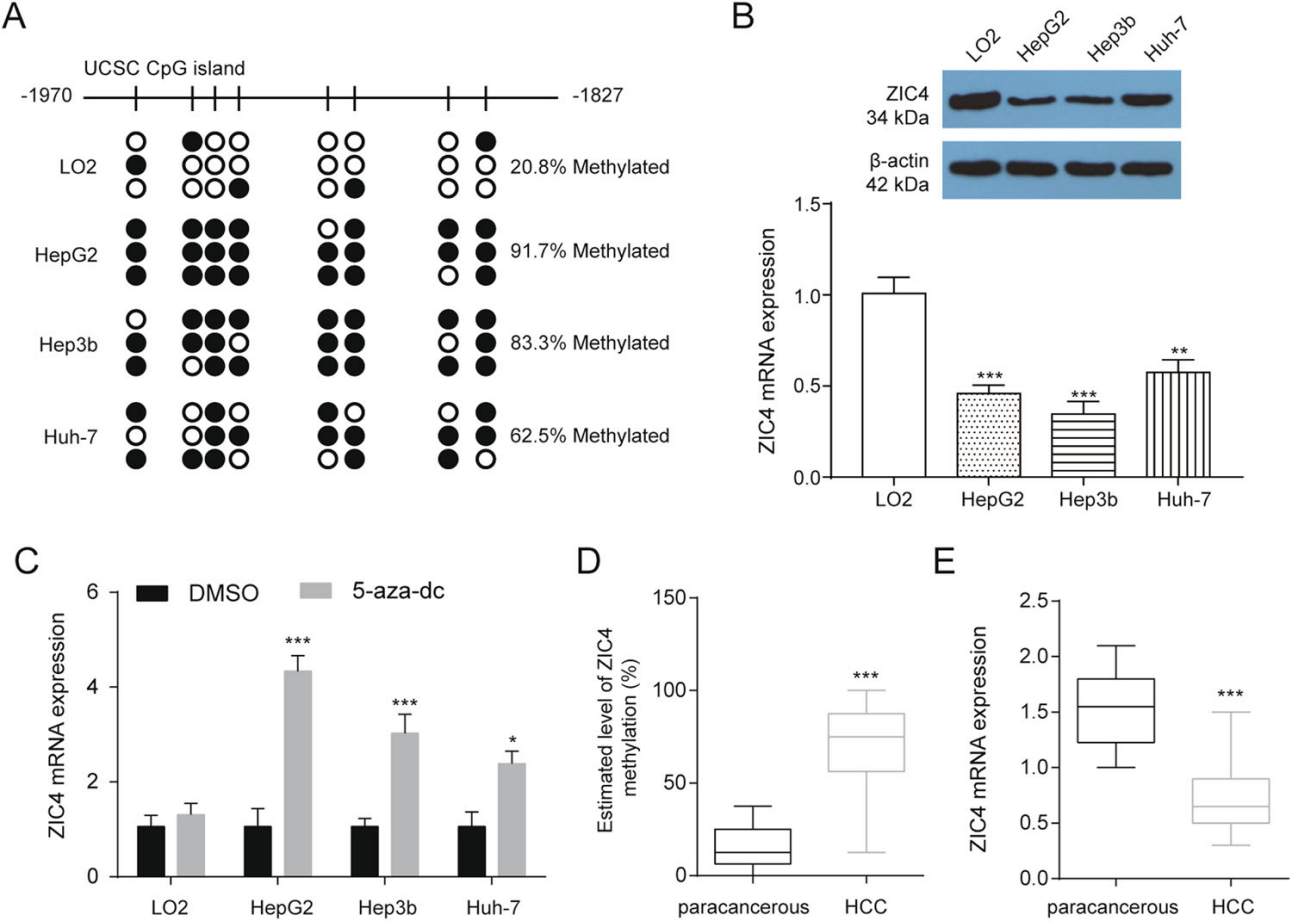
Methylation of *ZIC4* promoter and expression level in various liver cells. **A** The methylation status of the *ZIC4* promoter in LO2, HepG2, Hep3b, and Huh7 cell lines. The open and filled circles indicate the unmethylated and methylated CpGs, respectively (*n* = 3). **B** The protein and mRNA levels of *ZIC4* in LO2, HepG2, Hep3b, and Huh7 cell lines (*n* = 3). **P < 0.01, ***P < 0.001 vs. LO2. **C** *ZIC4* mRNA expression in indicated cells treated with 5 μmol 5-aza-dC for 72 h (*n* = 3). *P < 0.05, ***P < 0.001 vs. DMSO. **D** The methylation status of the *ZIC4* promoter in 20 pairs of HCC and paracancerous tissues. ***P < 0.001 vs. paracancerous. **E** The expression of the *ZIC4* mRNA in 20 pairs of HCC and paracancerous tissues. ***P < 0.001 vs. paracancerous.

We next investigated the potential molecular mechanism that mediates the downregulation of *ZIC4* in HCC. There was a typical CpG island in The *ZIC4* promoter, suggesting a possible involvement of DNA methylation in the regulation of *ZIC4*. Although 20.8% of CpGs were methylated in LO2 cells, 62.5–91.7% CpGs were methylated in HepG2, Hep3b, and Huh7 cells (Fig. 1A). The protein and mRNA levels of *ZIC4* in HepG2, Hep3b and Huh7 cell lines were significantly downregulated (Fig. 1B). The *ZIC4* expression was significantly increased when hepatoma cells with hypermethylated *ZIC4* promoter (HepG2, Hep3b, and Huh7) were treated with 5-aza-Dc for 3 days (Fig. 1C). Furthermore, *ZIC4* methylation was assessed by bisulfite-sequencing PCR (BSP) in 20 pairs of HCC and paracancerous tissues and ZIC4 was found to be hypermethylated in tumors tissues (Fig. 1D). ZIC4 expression was found to be downregulated in tumors tissues compared with paracancerous tissues (Fig. 1E).

### EZH2‑mediated H3K27me3 was involved in the repression of *ZIC4* in HCC cell lines

**Fig. 2:**
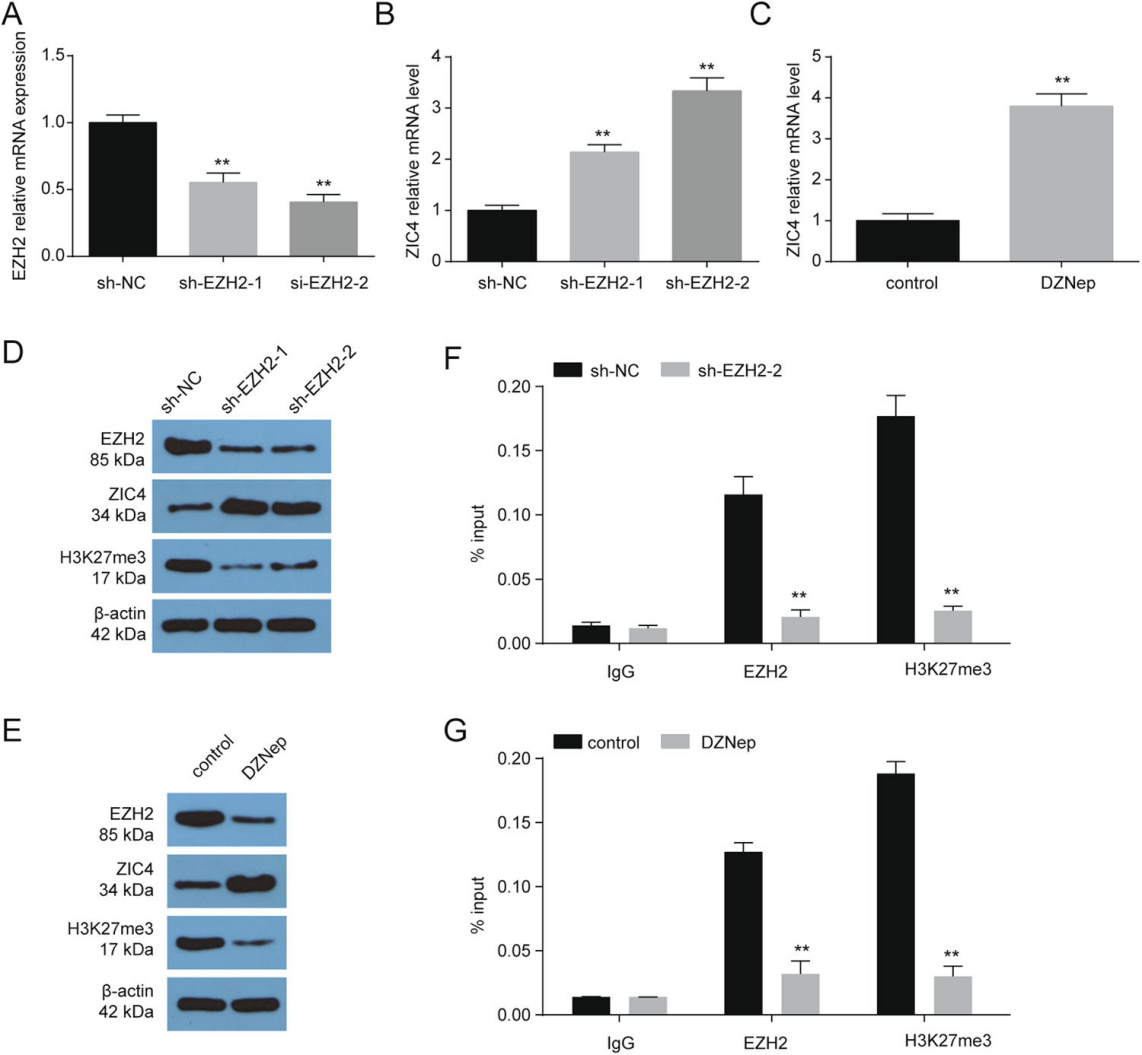
Expression of ZIC4 is modulated with EZH2 in HCC cells. **A** The expression of the *EZH2* mRNA in HepG2 without or with EZH2 sh-RNA treatment (*n* = 3). **P < 0.01 vs. sh-NC. **B** The expression of the *ZIC4* mRNA in HepG2 without or with EZH2 sh-RNA treatment (*n* = 3). **P < 0.01 vs. sh-NC. **C** The expression of the *ZIC4* mRNA in HepG2 without or with DZNep (10 μM) treatment for 72 h (*n* = 3). **P < 0.01 vs. control. **D** Western blot analysis of EZH2, ZIC4, H3K27me3, and β-actin in HepG2 without or with EZH2 sh-RNA treatment (*n* = 3). **E** Western blot analysis of EZH2, ZIC4, H3K27me3, and β-actin in HepG2 without or with DZNep (10 μM) treatment for 72 h (*n* = 3). **F** ChIP–qPCR analysis of EZH2 and H3K27me3 enrichment at upstream of *ZIC4* in HepG2 cells without or with EZH2 sh-RNA treatment (*n* = 3). **P < 0.01 vs. sh-NC. **G** ChIP–qPCR analysis of EZH2 and H3K27me3 enrichment at upstream of *ZIC4* in HepG2 without or with DZNep (10 μM) treatment for 72 h (*n* = 3). **P < 0.01 vs. control.

Epigenetic abnormalities of gene could also be associated with the acquisition of histone modifications, especially those of EZH2-dependent H3K27me3, may contribute to *ZIC4* repression. As expected, *ZIC4* mRNA expression was obviously upregulated in HepG2 cells treated with the sh-RNA of EZH2 and the specific inhibitor of EZH2, DZNep (Fig. 2A–C). Furthermore, sh-EZH2 and DZNep treatment led to a decrease in the levels of H3K27me3 in HepG2 cells (Fig. 2D, E). What’s more, ChIP assays showed that EZH2 occupied in the upstream region of ZIC4, which is concomitant with the increase in H3K27me3 levels (Fig. 2F, G). These data indicate a link between epigenetic regulation and ZIC4 transcription in hepatoma cell lines.

### ZIC4 mediate the effects of EZH2 on tumor promotion in vitro

**Fig. 3:**
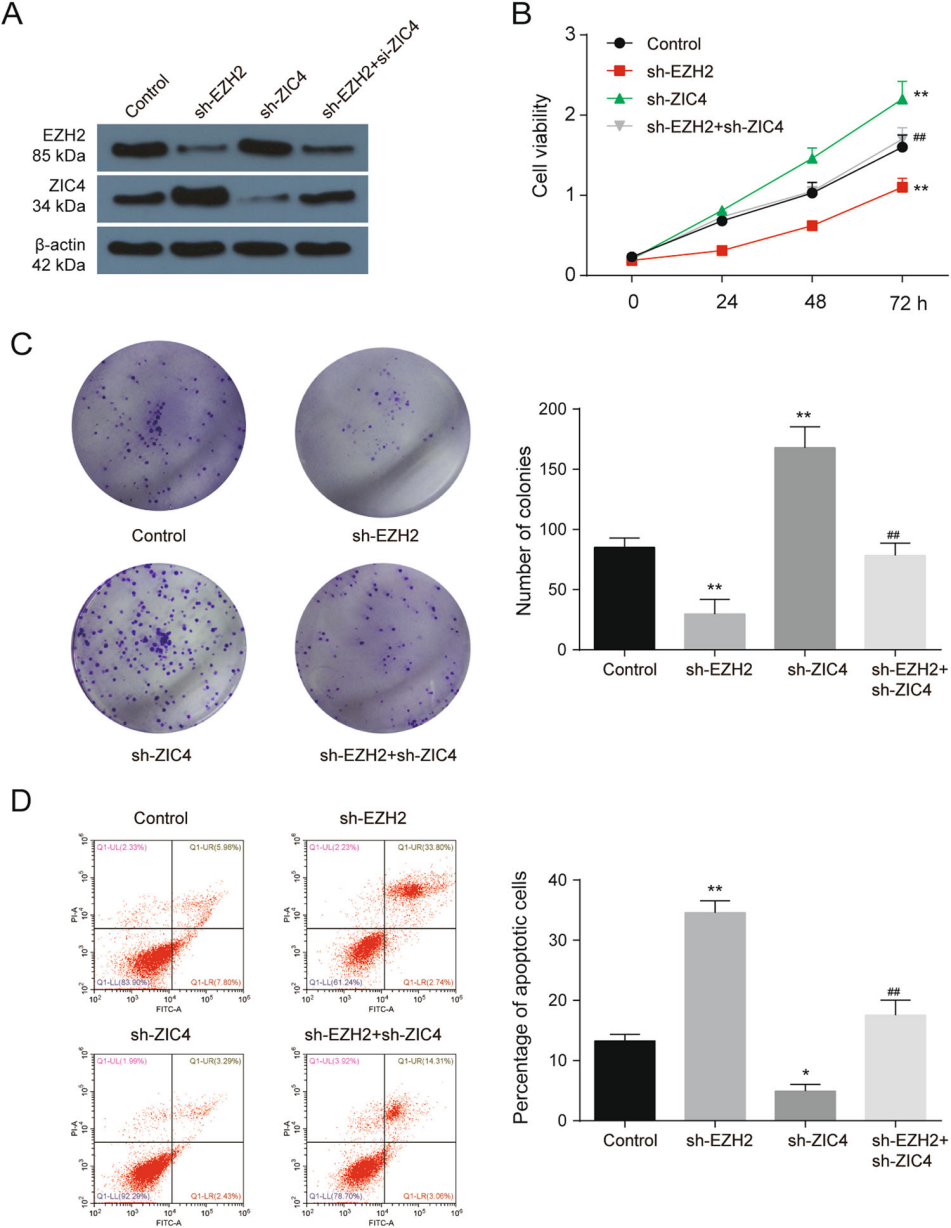
ZIC4 mediate the effects of EZH2 on tumor promotion in vitro. **A** EZH2 and ZIC4 protein levels in HepG2 cells with or without sh-EZH2 and sh-ZIC4 treatment (*n* = 3). **B** CCK-8 assay was performed to detected cell viability of HepG2 cells with or without sh-EZH2 and sh-ZIC4 treatment (*n* = 5). **C** Colony formation assay for HepG2 cells with or without sh-EZH2 and sh-ZIC4 treatment (*n* = 3). **D** The apoptosis of HepG2 cells with or without sh-EZH2 and sh-ZIC4 treatment (*n* = 3). *P < 0.05, **P < 0.01 vs. control; ## *P* < 0.01 vs. sh-EZH2.

**Fig. 4:**
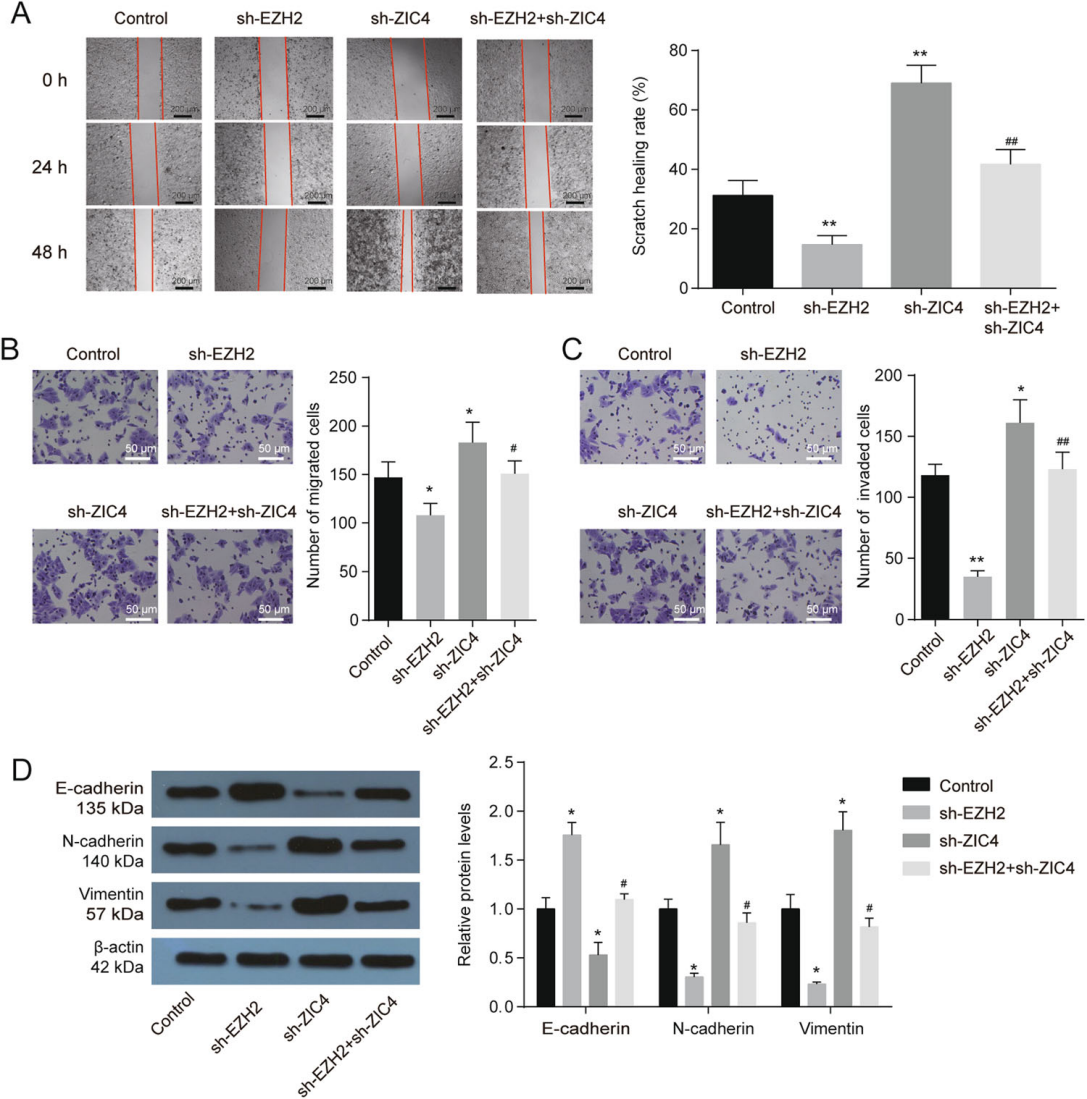
ZIC4 mediate the effects of EZH2 on tumor migration, invasion and EMT in vitro. **A** Wound Healing assays for HepG2 cells with or without sh-EZH2 and sh-ZIC4 treatment (*n* = 3). **B** Transwell assay was performed to detected cell migration ability for HepG2 cells with or without sh-EZH2 and sh-ZIC4 treatment (*n* = 3). **C** Transwell assay was performed to detected cell invasion ability for HepG2 cells with or without sh-EZH2 and sh-ZIC4 treatment (*n* = 3). **D** EMT relative protein levels were detected in HepG2 cells with or without sh-EZH2 and sh-ZIC4 treatment (*n* = 3). *P < 0.05, **P < 0.01 vs. control; ^#^*P* < 0.01, ^##^*P* < 0.01 vs. sh-EZH2.

To verify the roles of ZIC4 in vitro, CCK-8, anchorage‑dependent colony formation, flow cytometry, and transwell assays were employed. ZIC4 overexpression significantly inhibited HepG2 growth and survival as indicated by the reduction of cell viability and the decrease in the number of cloned cells (Fig. S2A–C). ZIC4 upregulation also was found to promote HepG2 apoptosis (Fig. S2D) and restrain the migration and invasion of HepG2 (Fig. S2E, F). What’s more, we also confirmed that DZNep could suppress HepG2 growth, clone formation, migration, and invasion in vitro (Fig. S3). To further explore the effects of EZH2 and ZIC4, HepG2 cells were co-transfected with or without sh-EZH2 and sh-ZIC4. The protein levels of EZH2 and ZIC4 in HepG2 with or without sh-EZH2 and sh-ZIC4 treatment suggesting that EZH2 knockdown promoted ZIC4 expression and ZIC4 knockdown did not affect EZH2 expression (Fig. 3A). Sh-EZH2 inhibited cell growth and promoted apoptosis, while sh-ZIC4 promoted the ability of growth and inhibited apoptosis. In addition, ZIC4 knockdown prevented the antitumor effect induced by sh-EZH2 (Fig. 3B–D). We next investigated the effect of EZH2 and ZIC4 on metastasis of HepG2 cells in vitro. Wound healing and transwell assays suggested that sh-EZH2 inhibited cell migration and invasion, while sh-ZIC4 promoted cell migration and invasion. ZIC4 knockdown rescued the antitumor effect induced by sh-EZH2 (Fig. 4A–C). What’s more, ZIC4 knockdown also rescued the inhibition of EMT progression induced by sh-EZH2, as the protein levels change of E-cadherin, N-cadherin, and vimentin (Fig. 4D).

### ZIC4 mediate the effects of DZNep on tumor promotion in vivo

**Fig. 5:**
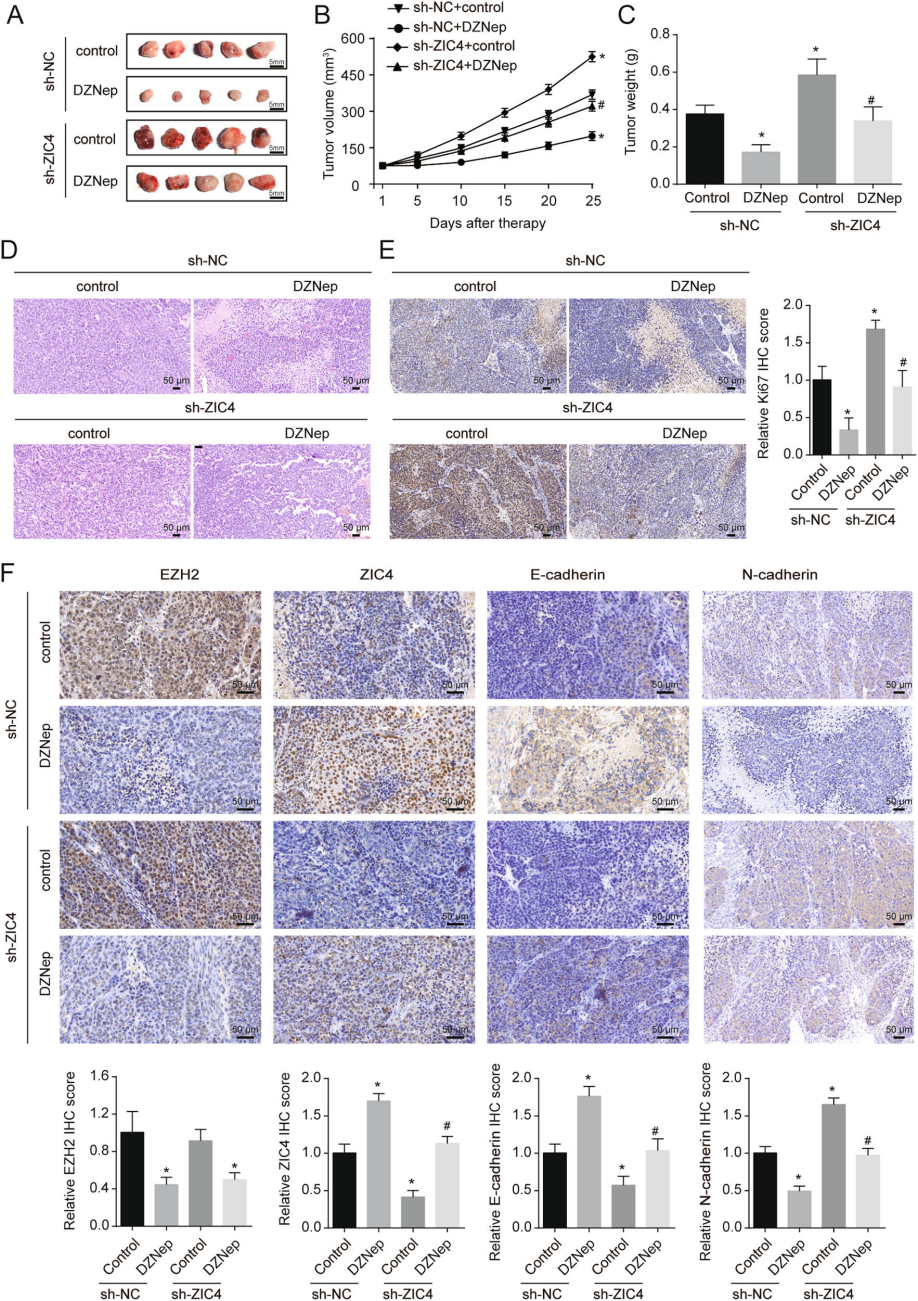
ZIC4 mediate the effects of DZNep on tumor promotion in vivo. The HCC Xenograft transplantation mice models were built with HepG2 with or without ZIC4 knock down. 1 week after bearing, DZNep (1 mg/Kg) was administered intraperitoneally twice per week and every 2 weeks. **A** The representative image of xenograft tumors that developed in nude mice injected subcutaneously with sh-NC or sh-HepG2 stable cells (*n* = 5). **B**, **C** Tumor growth curve and quantification of weight of the solid tumor after indicated treatment (*n* = 5). **D** Representative picture of HE staining for solid tumor (*n* = 5). **E** Representative picture of Ki-67 IHC staining for solid tumor (*n* = 5). **F** Representative picture of EZH2, ZIC4, E-cadherin, and N-cadherin IHC staining for solid tumor (*n* = 5). *P < 0.05 vs. sh-NC mice without DZNep treatment, ^#^*P* < 0.05 vs. sh-ZIC4 mice with DZNep treatment.

**Fig. 6:**
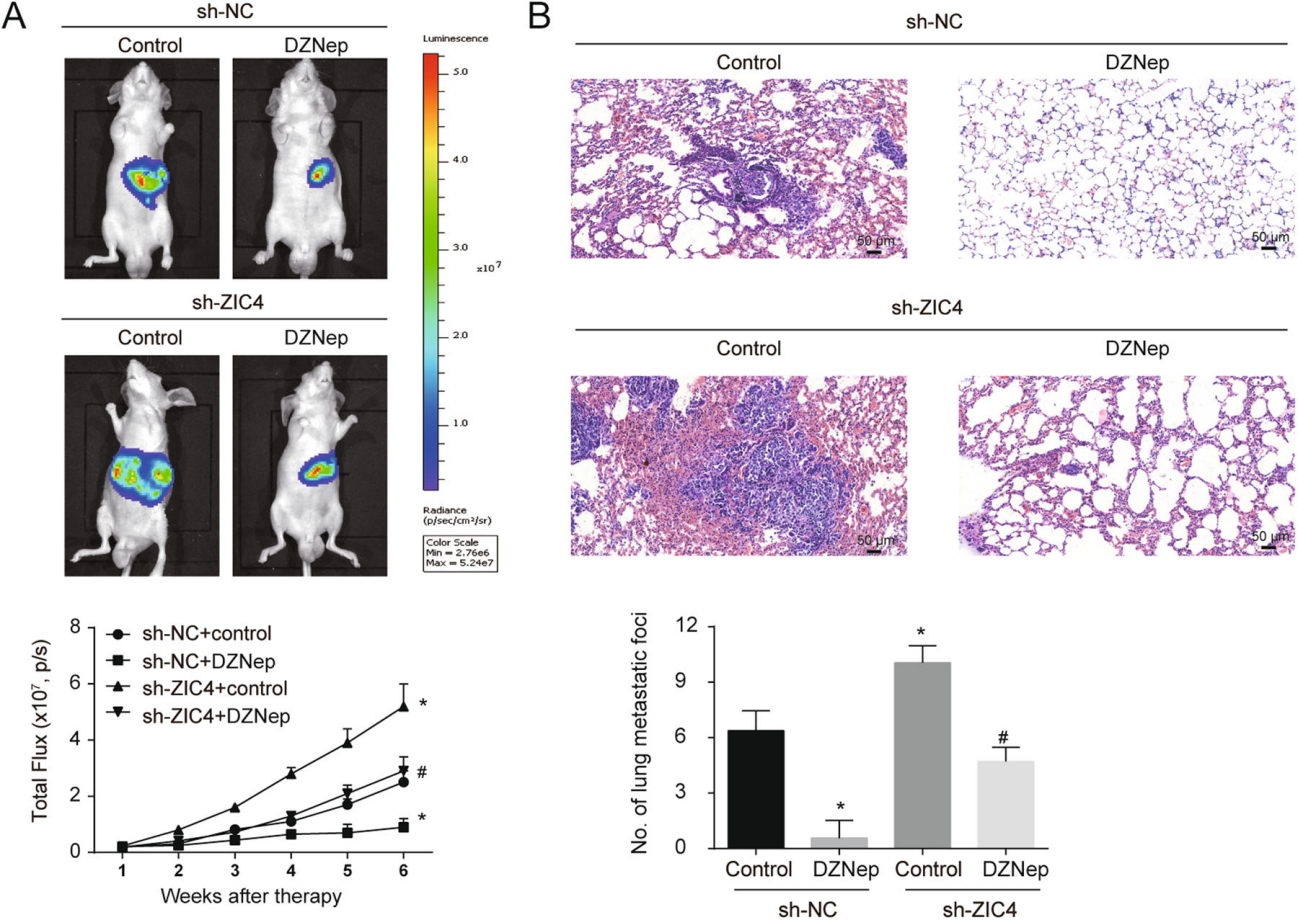
ZIC4 mediate the effects of DZNep on tumor pulmonary metastasis in vivo. The HCC orthotopic implantation mice models were built with HepG2 with or without ZIC4 knock down labeled by luciferase. 1 week after operation, DZNep (1 mg/Kg) was administered intraperitoneally twice per week and every 2 weeks. The growth of tumor was monitored weekly by IVIS lumina imaging system for 6 weeks. **A** Images of IVIS showed the condition of tumors in 6 weeks and the growth curve showed the total flux of tumors in each group every week detected by IVIS (*n* = 5). **B** Images showed the metastatic nodule in harvested lung tissues (*n* = 5). *P < 0.05 vs. sh-NC HepG2 stable cells without DZNep treatment, ^#^*P* < 0.05 vs. sh-ZIC4 HepG2 stable cells with DZNep treatment.

We next investigated the effect of EZH2 and ZIC4 on tumorigenesis and metastasis in vivo. Quantification of tumor size and weight showed that HepG2 cells treated sh-ZIC4 generated larger tumors and HepG2 cells treated DZNep generated smaller tumors. Downregulation of ZIC4 rescued the inhibition on HepG2 cells growth induced by DZNep in vivo (Fig. 5A–C). HE staining showed more necrotic area after DZNep treatment in sh-NC or sh-ZIC4 HepG2 xenograft (Fig. 5D) and Ki67 staining also indicated lesser Ki67 positive cell after DZNep treatment in sh-NC or sh-ZIC4 HepG2 xenograft (Fig. 5E). DZNep treatment inhibited EZH2 and N-cadherin expression and promoted ZIC4 and E-cadherin expression in solid tumor (Fig. 5F). ZIC4 knockdown rescued the repressive effect of DZNep on EMT progression. In addition, for HCC orthotopic implantation mouse models, in vivo imaging system (IVIS) showed that tumor growth was significantly suppressed after DZNep treatment and tumor growth was significantly promoted after ZIC4 knockdown (Fig. 6A). H&E staining of lung tissues also showed fewer and smaller metastatic nodules in DZNep group and showed more and larger metastatic nodules in sh-ZIC4 group (Fig. 6B). ZIC4 knockdown rescued the antitumor effect induced by DZNep in vivo. The above data show that inhibitory effects of DZNep were partially mediated by sh-ZIC4 treatment.

## Discussion

In this study, we explored the role of EZH2 and ZIC4 on the physiology and regulation in HCC and found that epigenetic silencing of *ZIC4* by EZH2 mediated H3K27me3 was an important mechanism in human liver cancer and it would provide a new therapeutic target for the treatment of hepatocellular carcinoma disease.

In the present study, we found that *ZIC4* was hypermethylated and downregulated in hepatocellular carcinoma cancer tissues and cells. EZH2 knockdown and DZNep resulted in low expressed of H3K27me3 while increased ZIC4 expression. The effects of EZH2 were partially mediated by ZIC4 treatment on HCC growth and metastasis in vitro and in vivo. Our study indicated that epigenetic silencing of *ZIC4* by EZH2 mediated H3K27me3 is an important mechanism in human liver cancer.

Many studies have confirmed the effect of CpG island hypermethylation on the regulation of cancer-associated genes in tumorigenesis and progression^17^. For example, the methylation degree of miR-608 was higher in two cancer cells lines compared with normal cell line with the methylation level of 91.4% and 87.3%, respectively, and also proposed that CpG islands hypermethylation might cause the silencing of mRNA expression^18^. In addition, Zhou et al. implicated that *DBCCR1* downregulation acted as an underlying module via DNA methylation in the lung cancer pathogenesis^19^. However, it was reported that the neural transcription factors *ZIC1* and *ZIC4* were upregulated in desmoid tumors and other fibroproliferative disorder diseases, the promoter performed unmethylated in tumor tissues compared to normal tissues^20^. In the present study, we found *ZIC4* was hypermethylated in HCC patients and cells, and the DNA methylation was responsible for the lower expressed mRNA, which was consistent with previous studies.

Recently, in order to decrease the methylation status of tumor inhibitor genes, epigenetic drugs are widely used to treat solid tumors with the advances of epigenetics knowledge^21^. Both DZNep treatment and EZH2 knockdown impeded anchorage-independent sphere formation and cell growth of HCC cells in culture, also pointed that and the tumor-initiating HCC cells are highly dependent on EZH2 for the tumorigenic activity^13^. Similarly, in our study, we found that EZH2 knockdown inhibited cell proliferation, migration, and promoted cell apoptosis and EZH2‑mediated H3K27me3 was involved in the repression of *ZIC4* in HCC cell lines.

As we all known that there are five members in *ZIC* gene family and each is responsible for encoding the zinc finger transcription factors, recently the mutant analysis in mice has proved the protection effect of these genes^22^. For example, *ZIC1* could act as a tumor inhibitor gene and suppressed cell proliferation via inactivating p-Erk1/2 and p-Akt pathway^23^. In addition, it is also revealed the tumor inhibition effect of *ZIC1* in malignant pleural mesothelioma cells^24^. Furthermore, a recent study displayed that *ZIC4* was involved with the development of tumors and patients with *ZIC4* hypermethylation status usually have shorter survival rate^16^. Consistent with the previous reports, we revealed that ZIC4 knockdown could promote cell proliferation, migration and inhibit cell apoptosis in vitro and promote the growth and metastasis in vivo.

In conclusion, the results of our study showed that *ZIC4* is hypermethylated and downregulated in HCC tissues and cell lines. EZH2‑mediated H3K27me3 was involved in the repression of *ZIC4* in HCC cell lines for the first time, which provided a new therapeutic target for the treatment of hepatocellular carcinoma disease.

## Supplementary information

Supplementary Figure 1

Supplementary Figure 2

Supplementary Figure 3

Supplementary Table S1

Supplementary Figure legends
