# Supplementary figures and images for: Epigenetic silencing of ZIC4 contributes to cancer progression in hepatocellular carcinoma
Source: Cell Death Dis. 2020 Oct 23;11(10):906. doi: 10.1038/s41419-020-03109-1 (PMC7584641; doi:10.1038/s41419-020-03109-1)

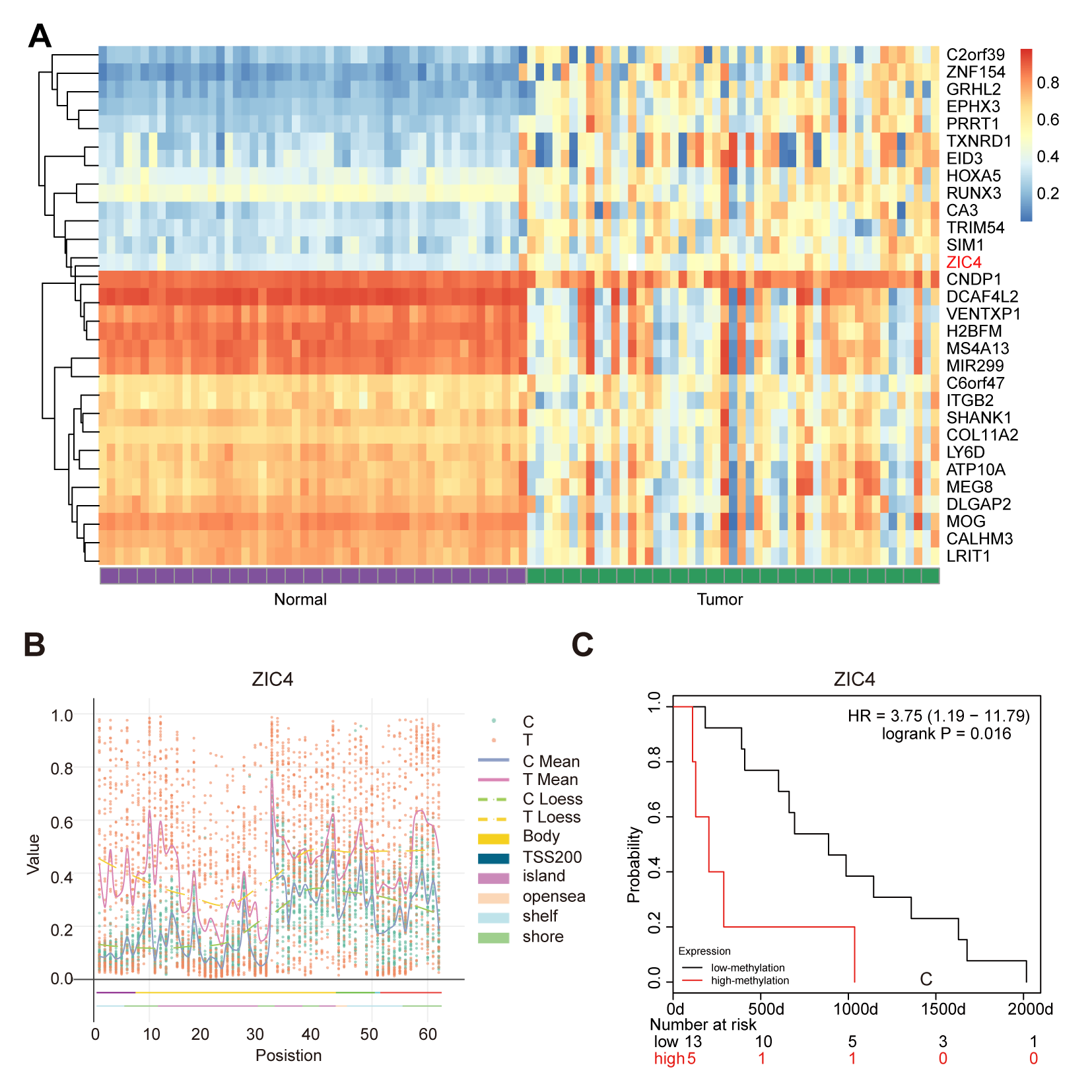

Supplement: Supplementary file 1 — Supplementary Figure 1 [file 41419_2020_3109_MOESM1_ESM.tif]

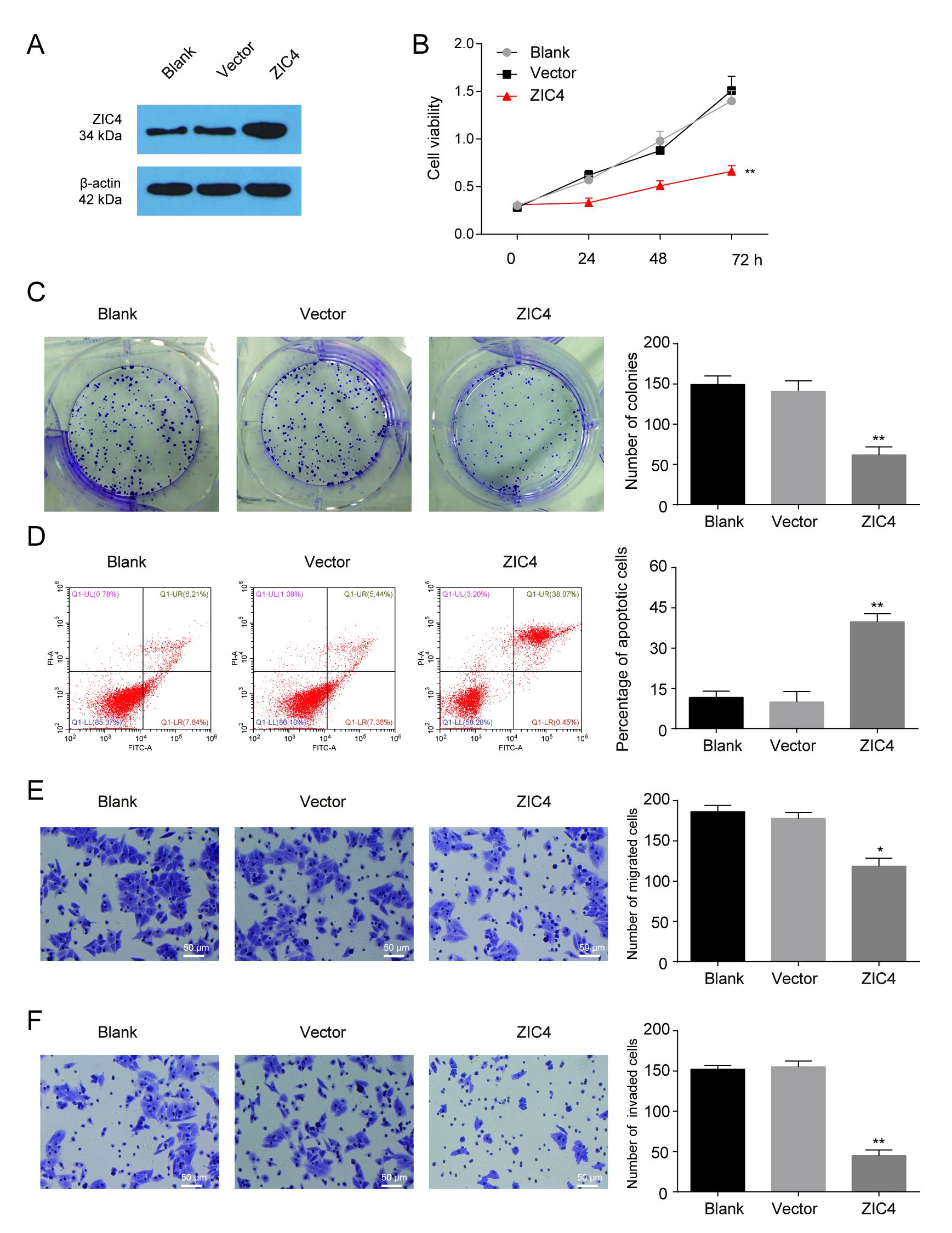

Supplement: Supplementary file 2 — Supplementary Figure 2 [file 41419_2020_3109_MOESM2_ESM.tif]

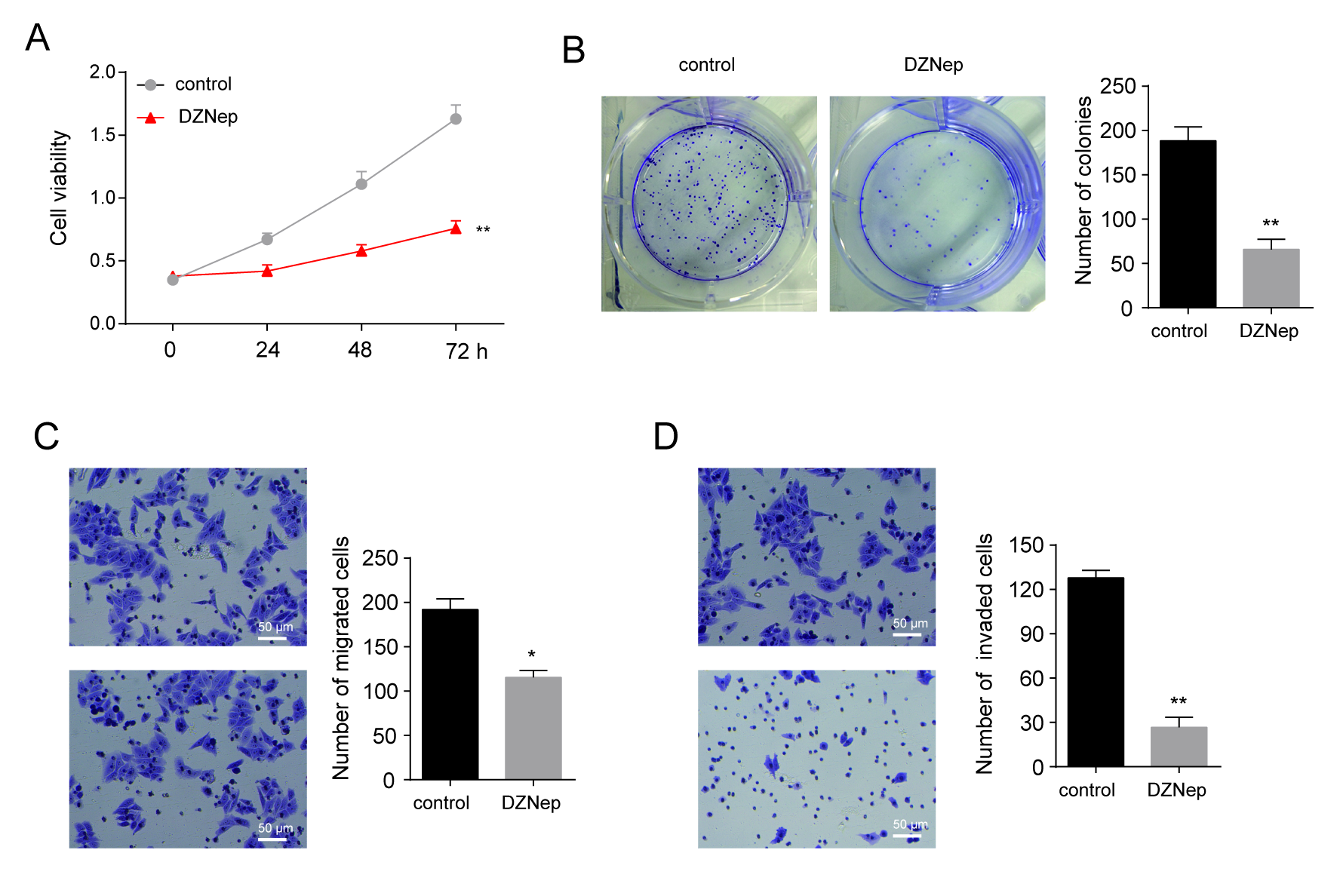

Supplement: Supplementary file 3 — Supplementary Figure 3 [file 41419_2020_3109_MOESM3_ESM.tif]
