# Supplementary material for: Epigenetic silencing of ZIC4 contributes to cancer progression in hepatocellular carcinoma
Source: Cell Death Dis. 2020 Oct 23;11(10):906. doi: 10.1038/s41419-020-03109-1 (PMC7584641; doi:10.1038/s41419-020-03109-1)
Supplement: Supplementary file 4 — Supplementary Table S1 [file 41419_2020_3109_MOESM4_ESM.docx]

**Supplementary Table S1. The sequence for qRT-PCR and sh-RNA.**

| Primers | Sequence (5’-3’) |
| --- | --- |
| EZH2-Forward | TAATGTGCTGGAATCAAAGGATAC |
| EZH2-Reverse | GCTTCATCTTTATTGGTGTTTGAC |
| ZIC4-Forward | CTAGCGACAAGCCATACACG |
| ZIC4-Reverse | GTAGCCGAATCGTAGCCAGA |
| GAPDH-Forward | TCAAGAAGGTGGTGAAGCAGG |
| GAPDH-Reverse | ATGATGACCCTTTTGGCTCC |
| Sh-EZH2-1 | CAGCUCUAGACAACAAACC |
| Sh-EZH2-2 | ACAGAAGAGGGAAAGUGUA |
| Sh-ZIC4 | AUAUAUUUACAAGAUCAAGCC |
| Sh-NC | CUUGAUCUUGUAAAUAUAUAA |
