# Supplementary material for: Epigenetic silencing of ZIC4 contributes to cancer progression in hepatocellular carcinoma
Source: Cell Death Dis. 2020 Oct 23;11(10):906. doi: 10.1038/s41419-020-03109-1 (PMC7584641; doi:10.1038/s41419-020-03109-1)
Supplement: Supplementary file 5 — Supplementary Figure legends [file 41419_2020_3109_MOESM5_ESM.docx]

**Supplementary Figure legends**

**Supplementary Figure S1 DNA methylation analysis of tumor tissues and normal tissues.**

(A) The top 30 candidate genes methylation degree were displayed, *ZIC4* was found to be hypermethylated in tumor tissues compared to paired normal tissues. (B) *ZIC4* was differentially methylated obvious among the top 30 candidate genes through analysis. (C) Patients in the ZIC4 high-methylation group had shorter overall survival by Kaplan-Meier.

**Supplementary Figure S2. ZIC4 overexpression inhibited HepG2 cell growth, survival, migration and invasion.**

(A) ZIC4 protein levels in HepG2 cells with or without ZIC4 upregulation (n=3). (B) CCK-8 assay was performed to detected cell viability of HepG2 cells with or without ZIC4 upregulation (n=5). (C) Colony formation assay for HepG2 cells with or without ZIC4 upregulation. (D) The apoptosis of HepG2 cells with or without ZIC4 upregulation (n=3). (E) Transwell assay was performed to detected cell migration ability for HepG2 cells with or without ZIC4 upregulation (n=3). (F) Transwell assay was performed to detected cell invasion ability for HepG2 cells with or without ZIC4 upregulation (n=3). **P*<0.05, ***P*<0.01 vs. vector.

**Supplementary Figure S3.** **DZNep inhibited HepG2 cell growth, clone formation, migration and invasion.**

(A) CCK-8 assay was performed to detected cell viability of HepG2 cells with or without DZNep treatment (n=5). (B) Colony formation assay for HepG2 cells with or without DZNep treatment (n=3). (C-D) Transwell assay was performed to detected cell migration and invasion ability for HepG2 cells with or without DZNep treatment (n=3). **P*<0.05, ***P*<0.01 vs. control.
